# Supplementary material for: Effect of High-Density Polyethylene Microplastics on the Survival and Development of Eastern Oyster (Crassostrea virginica) Larvae
Source: Int J Environ Res Public Health. 2023 Jun 16;20(12):6142. doi: 10.3390/ijerph20126142 (PMC10298504; doi:10.3390/ijerph20126142)
Supplement: Supplementary file 1 [file ijerph-20-06142-s001.zip › ijerph-2283968-supplementary.pdf]

Table S1: Survival rates of *C. virginica* after HDPE-MPs exposure in Cohort 1

day	Treatment	Survival rate
3	control	64.83
3	control	51.8
3	mp	37
3	mp	35
5	control	19.96
5	control	21.45
5	mp	37.41
5	mp	55.21
7	control	57.1
7	control	66.5
7	mp	53.31
7	mp	47.5
9	control	3.78
9	control	18.6
9	mp	24.8
9	mp	19.9

Table S2: Survival rates of *C. virginica* after HDPE-MPs exposure in Cohort 2

day	Treatment	Survival rate
4	control	35.5
4	control	31.2
4	mp	31.2
4	mp	32
9	control	11.28
9	control	15.38
9	mp	8.55
9	mp	8.33
11	control	11
11	control	18.33
11	mp	0
11	mp	0

Table S3: Survival rates of *C. virginica* after HDPE-MPs exposure in Cohort 3

day	Treatment	Survival rate
4	control	76.87
4	control	80.83
4	mp	84.22
4	mp	98.33
6	control	38.83
6	control	30.92
6	mp	56.13
6	mp	50.84
7	control	39.75
7	control	73.33
7	mp	61.54
7	mp	62.22
10	control	16.98
10	control	33.34
10	mp	50
10	mp	14.29
